# PD-L1 and PD-L2 Expression in Cervical Cancer: Regulation and Biomarker Potential

**DOI:** 10.3389/fimmu.2020.596825

**Published:** 2020-12-17

**Authors:** Jossie Rotman, Leontine A. S. den Otter, Maaike C. G. Bleeker, Sanne S. Samuels, A. Marijne Heeren, Margaretha G. M. Roemer, Gemma G. Kenter, Henry J. M. A. A. Zijlmans, Nienke E. van Trommel, Tanja D. de Gruijl, Ekaterina S. Jordanova

**Affiliations:** ^1^ Center for Gynecologic Oncology Amsterdam (CGOA), Amsterdam University Medical Center (UMC), Cancer Center Amsterdam (CCA), Vrije Universiteit Amsterdam, Amsterdam, Netherlands; ^2^ Department of Medical Oncology Amsterdam UMC, Cancer Center Amsterdam (CCA), Vrije Universiteit Amsterdam, Amsterdam, Netherlands; ^3^ Department of Pathology, Cancer Center Amsterdam (CCA), Amsterdam UMC, Vrije Universiteit Amsterdam, Amsterdam, Netherlands; ^4^ Center for Gynecologic Oncology Amsterdam (CGOA), Netherlands Cancer Institute—Antoni van Leeuwenhoek (NKI-AVL), Amsterdam, Netherlands; ^5^ Department of Pathology, Leiden University Medical Center, Leiden, Netherlands

**Keywords:** cervical cancer, programmed cell death ligand 1, programmed cell death ligand 2, fluorescence *in situ* hybridization, RNAish, immunohistochemistry, The Cancer Genome Atlas

## Abstract

PD-1/PD-L1 immune checkpoint inhibitors show potential for cervical cancer treatment. However, low response rates suggest that patient selection based on PD-L1 protein expression is not optimal. Here, we evaluated different PD-L1 detection methods and studied transcriptional regulation of PD-L1/PD-L2 expression by The Cancer Genome Atlas (TCGA) mRNAseq analysis. First, we determined the copy number of the PD-L1/PD-L2 locus by fluorescence *in situ* hybridization (FISH), PD-L1 mRNA expression by RNA *in situ* hybridization (RNAish), and PD-L1/PD-L2 protein expression by immunohistochemistry (IHC) on tissue microarrays containing a cohort of 60 patients. Additionally, distribution of PD-L1/PD-L2 was visualized based on flow cytometry analysis of single-cell suspensions (n = 10). PD-L1/PD-L2 locus amplification was rare (2%). PD-L1 mRNA expression in tumor cells was detected in 56% of cases, while 41% expressed PD-L1 protein. Discordant scores for PD-L1 protein expression on tumor cells between cores from one patient were observed in 27% of cases. Interestingly, with RNAish, PD-L1 heterogeneity was observed in only 11% of the cases. PD-L2 protein expression was found in 53%. PD-L1 mRNA and protein expression on tumor cells were strongly correlated (p < 0.001). PD-L1 and PD-L2 protein expression showed no correlation on tumor cells (p = 0.837), but a strong correlation on cells in stromal fields (p < 0.001). Co-expression of PD-L1 and PD-L2 on macrophage-like populations was also observed with flow cytometry analysis. Both PD-L1 and PD-L2 TCGA transcript levels strongly correlated in the TCGA data, and both PD-L1 and PD-L2 strongly correlated with interferon gamma (IFNG) expression/transcript levels (p < 0.0001). Importantly, patients with high PD-L1/PD-L2/IFNG transcript levels had a survival advantage over patients with high PD-L1/PD-L2 and low IFNG expression. Based on these findings, we conclude that PD-L1/PD-L2 expression in cervical cancer is mainly associated with interferon induction and not gene amplification, which makes FISH unsuitable as biomarker. The heterogeneous PD-L1 and PD-L2 expression patterns suggest IHC unreliable for patient selection. RNAish, in conjunction with interferon signaling evaluation, seems a promising technique for immune checkpoint detection. These results warrant further investigation into their prognostic and predictive potential.

## Introduction

Cervical cancer is the second most common gynecologic tumor and a leading cause of cancer-related death for women worldwide (1). It is caused by a persistent infection with high-risk types of the human papillomavirus (HPV) (2). Squamous cell carcinoma (SCC) and adenocarcinoma (AC) are the two most prevalent histological subtypes, with differences in terms of oncogenic mutations, immune microenvironment, response to treatment and disease outcome (3–7). Despite advancements in prevention and treatment over the last decades, the overall patient survival rate is still less than 60% in cases of regional disease, and < 20% if distant metastases are present (8). Therefore, alternative treatment options such as immunotherapy are currently explored.

Targeting of the PD-1/PD-L1 pathway has shown the most successful responses by immune checkpoint inhibition for various cancer types (9–12). Programmed cell death ligand 1 (PD-L1) is a receptor mainly expressed on tumor cells and various myeloid cell types (13). By binding to its receptor, programmed cell death protein 1 (PD-1) on activated T cells, the immune response is inhibited (14). PD-L2 is a less-studied ligand of PD-1 and mainly present on activated dendritic cells and macrophages, which play an important role in the inhibition of the anti-tumor immune response (15). The regulation mechanisms of PD-L1/PD-L2 expression are not fully understood. Genetic aberrations at the 9p24.1 chromosomal region, transcriptional regulation by factors such as interferons, oncogenic signaling pathways (e.g., JAK/STAT and RAS/ERK signaling), epigenetic modifications, and productive infection with HPV (in case of cervical cancer), may all play individual or synergistic roles in the regulation of PD-L1/PD-L2 (16–19).

The prognostic significance of PD-L1 protein expression in cervical cancer has been reported in a few studies with contradictory results (19–23). We have shown that not only the extent, but also the pattern of PD-L1 expression is an important prognostic factor, since marginal PD-L1 expression, most likely induced by interferon gamma (IFNG) signaling, was associated with favorable prognosis when compared to diffuse PD-L1 expression or lack of PD-L1 (24). The prognostic potential of PD-L2 has not been studied in cervical cancer, but was associated with worse prognosis in patients with other types of solid tumors (25).

Results of the phase II KEYNOTE-158 study demonstrated the efficacy of the PD-1 inhibitor pembrolizumab in cervical cancer which led to Food and Drug Administration (FDA) approval (26). The objective response rate was 12.2% (12/98) and no clinical activity was observed in patients with PD-L1-negative tumors, as assessed by immunohistochemistry (IHC). In another study with less heavily pre-treated patients with advanced cervical cancer (n = 19), an overall response rate (ORR) of 26% was observed after nivolumab (anti-PD-1) treatment, including a complete response in a patient lacking tumoral PD-L1 expression (27).

These data suggest that PD-L1 protein expression is not optimal as prognostic or predictive biomarker for response on treatment. Also in melanoma and lung cancer, treatment response was observed in PD-L1-negative tumors (28). Patient selection according to PD-L1 protein expression thereby excludes potential patients for whom anti-PD-(L)1 therapy could be effective. PD-L1 DNA and/or mRNA status, or inclusion of PD-L2 expression may provide more suitable predictive biomarkers for PD-(L)1 checkpoint therapy.

In this study, we evaluated different detection methods of PD-L1 and PD-L2 in cervical cancer on DNA, mRNA and protein level to address two major questions: 1.) What is the main mechanism leading to PD-L1/PD-L2 expression and 2.) Which molecular method should be used for detection of the notoriously heterogeneous expression patterns? Ultimately, our findings may also lead to the development of more reliable prognostic and predictive biomarkers.

## Materials and Methods

### Patients and Samples

Sixty patients with a pathologically confirmed diagnosis of cervical cancer who underwent radical hysterectomy or conisation/loop excision between 1991 and 2012 in the Netherlands Cancer Institute – Antoni van Leeuwenhoek (NKI-AVL) hospital, Amsterdam, were included. The use of data and material was approved by the Institutional Review Board (IRB; IRBd19076) of the NKI-AVL according to national regulations in The Netherlands concerning the proper use of clinical materials. Patient data were pseudonymized and clinicopathological characteristics were collected retrospectively from medical records. The International Federation of Gynecology and Obstetrics (FIGO) staging system from 2009 was used for the patient data. Patient characteristics are shown in **Table 1**. Mean age at diagnosis was 46 ± 12 years. Forty-four of 60 cases were histologically categorized as SCC, 14 cases as AC and 2 cases as adenosquamous cell carcinoma (ASCC). Patient characteristics showed no significant differences for SCC vs. AC subgroups (data not shown).

**Table 1:** Clinicopathological characteristics of the study population.

Clinicopathological characteristics		Total n (%)
**Number of patients**		**60**
Age at diagnosis in years (Mean ± SD)		46 ± 12
Histology	AC	14 (23)
	SCC	44 (73)
	ASCC	2 (4)
HPV type^a^	Type 16	28 (47)
	Type 18	14 (23)
	Other type	9 (15)
	Negative	4 (7)
FIGO stage (2009)	≤IB1	43 (72)
	>IB1	17 (28)
Tumor size^b^	≤40 mm	45 (75)
	>40 mm	13 (22)
Infiltration depth^c^	≤15 mm	44 (73)
	>15 mm	12 (20)
Vaginal involvement^d^	No	47 (78)
	Yes	12 (20)
Parametrium invasion^e^	No	54 (90)
	Yes	5 (8)
Lympho-vascular space invasion^f^	No	22 (37)
	Yes	24 (40)
Tumor-positive lymph nodes^g^	No	38 (63)
	Yes	18 (30)
Recurrence in 5 years	No	52 (87)
	Yes	8 (13)
Primary treatment^h^	Surgery	36 (60)
	Surgery + adjuvant tr.	21 (35)
	Chemoradiation	1 (2)
	Neo-adjuvant tr. + surgery	1 (2)

FIGO, International Federation of Gynecology and Obstetrics; SCC, squamous cell carcinoma; AC, adenocarcinoma; ASCC, adenosquamous cell carcinoma.

a Data missing for 5 cases.

b Data missing for 2 cases.

c Data missing for 4 cases.

d Data missing for 1 case.

e Data missing for 1 case.

f Data missing for 14 cases.

g Data missing for 4 cases.

h Data missing for 1 case.

This tissue was obtained after conization in a patient with stage IA1 disease.

Formalin-fixed, paraffin-embedded (FFPE) material was collected from primary cervical tumor samples and two or three core 4-mm biopsies were punched for tissue microarray (TMA) construction. A total of 178 FFPE core biopsies were divided over two TMAs.

Additionally, fresh cervical primary tumor samples were collected from 10 patients that underwent radical hysterectomy at the NKI-AVL or AmsterdamUMC and used for flow cytometry analysis, as described below. This study design was approved by the local IRB (no. NL25610.058.08) and patients gave written informed consent.

### FISH for Copy Number Analysis of the PD-L1/PD-L2 Locus (9p24.1)

Copy number analysis (CNA) of the PD-L1/PD-L2 locus was performed by fluorescent *in situ* hybridization (FISH) as previously described (29, 30). In short, DNA of bacterial artificial chromosome (BAC) clones (Source Bioscience, Nottingham, UK), extracted from Luria broth cultures with the Qiagen Maxi-prep Kit (Hilden, Germany) was used. The DNA was repeat depleted using Kreatech’s proprietary Repeat-Free technology and labeled using Kreatech’s (Leica Biosystems) proprietary ULS™ labeling. Platinum*Bright™*495 (green) targeting 9p24.1 which encompassed PD-L1 locus CD274, a Platinum*Bright*™550 (red) probe also targeting 9p24.1 and encompassing the PD-L2 locus PDCD1LG2 and a (blue) probe labeled with Platinum*Bright*™415 that targeted the SE9 (D9Z4) centromeric region. Slides were hybridized following the manufacturer’s recommendations (Leica Biosystems). Approximately 100 cells per core biopsy to a total of 200–300 cells per patient were analyzed manually on a Leica Biosystems DM5500B microscope, equipped with a DFC365FX camera. Cases were classified as “disomy” when nuclei had a target:control ratio of 1:1 and contained two red signals targeting PD-L1, two green signals targeting PD-L2 (target) and two blue signals targeting the centromeric region of chromosome 9 (control). In cases classified as “polysomy”, nuclei also had a target:control ratio of 1:1, but more than 2 copies. Cases were classified as “monosomy or deletion” when nuclei contained only one red (PD-L1) and green signal (PD-L2) and either one, or two blue control signals, respectively. “Gain” was scored when nuclei had a target:control ratio of > 1:1 but < 3:1. Cases were classified as “amplification” when nuclei had a target:control ratio ≥ 3:1 (29, 31).

### RNAish for Detection of PD-L1 mRNA Expression

RNAscope^®^ was used to detect and quantify PD-L1 mRNA molecules (32). The staining was performed by Pharma Assay Services at Advanced Cell Diagnostics (ACD), USA. TMAs were hybridized with RNAscope^®^ Probe Hs-CD274-C2, the positive control probe (Hs-PolR2a-C2) and negative control probe DapB (DapB-C2). RNA *in situ* hybridization (RNAish) signals were amplified and visualized by using the RNA ISH Detection kit – RED (ACD). Positive signals were visible as red punctate dots in cytoplasm and/or nucleus, with each signal corresponding to a single PD-L1 mRNA molecule. PD-L1 mRNA expression in tumor cells was scored by the mean number of dots per cell and by the percentage of PD-L1 mRNA positivity in tumor tissue (0: no dots; 1: 1–3 dots per tumor cell in <10% of tumor fields; 2: 1–3 dots per tumor cell in ≥ 10% of tumor fields; 3: 4–9 dots per tumor cell; 4: ≥10 dots per tumor cell). Cases were further classified as negative (score of 0) or positive (score of ≥1) for statistical analysis. PD-L1 mRNA expression in stromal compartment cells was scored as negative or positive. Slides were analyzed and imaged on a brightfield microscope (Olympus BX50; Olympus, Center Valley, PA, USA).

### Immunohistochemistry for Detection of PD-L1/PD-L2 Protein Expression

PD-L1/PD-L2 protein expression was detected by standard immunohistochemical DAB staining as previously described (4) using rabbit anti-PD-L1 antibody (1:100, SP‐142, Spring Bioscience, USA) and rabbit anti-PD-L2 antibody (1:200, D7U8C; Cell Signaling, USA). Standard negative and positive FFPE tonsil tissue control slides were included during the staining. Slides were deparaffinized in 3× xylene and washed in 1× 100%, 1× 90% of ethanol. Then, endogenous peroxidase was blocked with 0.3% H2O2 (MERCK, Germany) in methanol for 20 min. Slides were rehydrated in 1× 70% of ethanol and 1× demineralized water and heated in a microwave for antigen retrieval for 10 min in boiling Tris/EDTA buffer pH 9.0. The slides were allowed to cool down for 1 h at room temperature (RT). After antigen retrieval, all slides were washed with 2× demineralized water and 2× phosphate buffered saline (PBS) and incubated overnight at RT with the PD-L1 or PD-L2 antibodies. The next day, slides were washed 3× in PBS and incubated with BrightVision (ImmunoLogic, The Netherlands) for 30 min at RT. Then, slides were washed 3× in PBS, after which immune complexes were visualized using 3,3’-diaminobenzidine tetrahydrochloride (Sigma, USA). Slides were counterstained with Haematoxylin followed by 5 min rinsing with running tap water. Finally, sections were dehydrated and mounted under coverslips with Quick-D mounting medium (Klinipath, The Netherlands). Slides were analyzed and imaged on a brightfield microscope (Olympus BX50; Olympus, Center Valley, PA, USA) and scored by two individuals (EJ and LO) blinded to the clinical data. Haematoxylin nuclear staining was used to distinguish tumor fields from stromal tissue. PD-L1 and PD-L2 expression in tumor tissue was scored in *H score*; the sum of intensity (0: negative; 1: weak; 2: clear; 3: strong) and percentage (0: 0%–1%; 1: 1%–10%; 2: 10%–25%; 3: 25%–50%; 4: 50%–75%; 5: 75%–100%). For cells in stromal fields we scored expression in three categories: 0: negative; 1: weak; 2: strong. The mean expression of the core biopsies that were suitable for evaluation was used. If only one core was available, these cases were not evaluated and were left out of all following analyses.

### Flow Cytometry and High-Dimensional Analysis

For phenotypic analysis of myeloid cells in 7 SCC and 3 AC tumors, multi-color flow cytometry was carried out using the LSR Fortessa X-20 (BD). Samples were freshly collected as described previously (33, 34). A total of 200,000 cells per tumor sample were stained using the following directly labeled surface antibodies: CD1a-PE (1:50, BD), CD14-PerCP-Cy5.5 (1:20, BD), CD11c-APC (1:100, BD), CD1c-PE-Cy7 (1:100, Biolegend), CD45-AF700 (1:200, Biolegend), PD-L2-BV711 (1:25, BD), PD-L1-BV786 (1:25, BD), CD80-FITC (1:50, BD), and CD163-BV421 (1:70, BD). Data were visualized in t-Distributed Stochastic Neighbor Embedding (t-SNE) density plots generated in FCS express 6 (De Novo software).

### TCGA mRNA Expression Analysis

For additional analysis, publicly available mRNA sequencing and copy number data of The Cancer Genome Atlas (TCGA) database from cervical cancer patients (n = 299, SCC and AC only) was retrieved by using the ‘R2: Genomics Analysis and Visualization Platform (http://r2.amc.nl) and cBioPortal for Cancer Genomics Interface (https://cbioportal.org) (35, 36).

### Statistical Analyses

For statistical analyses IBM SPSS version 22 and GraphPad Prism version 8 was used. Continuous variables were reported as mean ± standard deviation (SD) and categorical variables as number (n) and percentage (%). The Pearson’s χ^2^ and/or Fisher’s exact tests was used to compare parameters between groups. The Kruskall-Wallis test was used to determine the relation between DNA-, mRNA-, and/or protein status. Kaplan-Meier survival curves and the Log-rank test were used to determine survival outcomes in the TCGA database. RStudio was used to generate a heat map with cluster analysis. Statistical significance was defined as p ≤ 0.05 two-sided.

## Results

### NKI-AVL Patient Cohort: Gene Locus Status and mRNA and Protein Expression of PD-L1 and PD-L2

In **Table 2**, the results of the gene status and mRNA- and protein expression analyses of PD-L1 and DNA- and protein expression analyses for PD-L2 are summarized.

**Table 2:** Gene status, RNA, and protein expression of PD-L1 and PD-L2.

PD-L1/2 DNA status		Total n (%)	SCC n (%)	AC n (%)	ASCC n (%)	*p-*value*^a^*
Disomy		43 (81)	32 (60)	10 (19)	1 (2)	0.862
Monosomy/deletion		5 (9)	5 (9)	0 (0)	0 (0)	
Polysomy		4 (8)	2 (4)	1 (2)	1 (2)	
Amplification		1 (2)	1 (2)	0 (0)	0 (0)	
**PD-L1 mRNA expression**		**Total** n (%)	**SCC**n (%)	**AC**n (%)	**ASCC**n (%)	*p-*value*^a^*
**Tumor cells**	Negative	24 (44)	15 (28)	8 (15)	1 (2)	0.226
	Positive	30 (56)	24 (44)	6 (11)	0 (0)
**Stromal cells^†^**	Negative	22 (42)	13 (25)	9 (17)	0 (0)	0.049
	Positive	30 (58)	25 (48)	4 (8)	1 (2)
**PD-L1 protein expression**		**Total** n (%)	**SCC**n (%)	**AC**n (%)	**ASCC**n (%)	*p-*value*^a^*
**Tumor cells**	Negative	35 (59)	23 (39)	11 (19)	1 (2)	0.124
	Positive	24 (41)	20 (34)	3 (5)	1 (2)
**Stromal cells^†^**	Negative	10 (17)	5 (9)	5 (9)	0 (0)	0.044
	Positive	48 (83)	37 (64)	9 (16)	2 (3)
**PD-L2 protein expression**		**Total** n (%)	**SCC**n (%)	**AC**n (%)	**ASCC**n (%)	*p-*value*^a^*
**Tumor cells**	Negative	28 (47)	14 (24)	13 (22)	1 (2)	0.000
	Positive	31 (53)	29 (49)	1 (2)	1 (2)
**Stromal cells^†^**	Negative	36 (61)	22 (37)	13 (22)	1 (2)	0.005
	Positive	23 (39)	21 (36)	1 (2)	1 (2)

SCC, squamous cell carcinoma; AC, adenocarcinoma; ASCC, adenosquamous cell carcinoma.

^a^Fisher’s exact or χ ^2^ and calculated only between SCC and AC, due to the limited number of ASCC cases.

^†^Cells within stromal compartment. Underlined means statistically significant.

**Figure 1:**
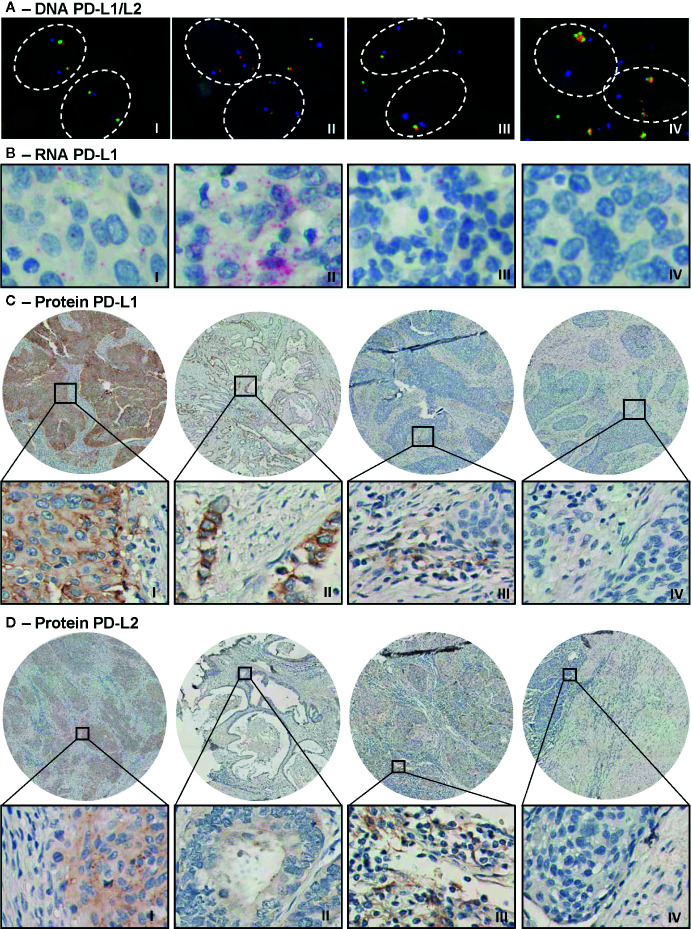
Representative microscopic images of PD-L1/PD-L2 DNA FISH, PD-L1 RNAish and immunohistochemical PD-L1 and PD-L2 protein expression. **(A)** Microscopic images of a PD-L1/PD-L2 disomy (I), polysomy (II), monosomy/heterozygous deletion (III), and amplification (IV) case. Visualization of CD274 (PD-L1 locus) in green, of PDCD1LG2 (PD-L2 locus) in red, and of the centromeric region in blue. **(B)** Moderate (I, score = 3) and strong (II, score = 4) PD-L1 mRNA expression (in red) in tumor cells. Expression of PD-L1 mRNA was also observed in cells in stroma (III). The right panel (IV) shows a negative tumor for PD-L1 mRNA. **(C)** Strong PD-L1 expression (in brown) on tumor cells (I, H-score = 8) in an SCC core biopsy and an example of intermediate expression (II, H-score = 5) in an AC tumor. The third image (III) shows positive cells in stroma and the last image is a negative tumor for PD-L1. **(D)** Strong PD-L2 expression (in brown) on tumor cells in an SCC sample (I, H-score = 8) and weak PD-L2 expression on tumor cells in AC (II, H-score = 2). Image III shows positive stromal fields for PD-L2 protein and the last image shows a negative tumor for PD-L2.

DNA status analysis by FISH showed that most of the evaluable cervical cancer cases had disomy of chromosome 9 with two signals for both the CD274 (PD-L1) and PDCD1LG2 (PD-L2) locus (n = 43, 81%). In 9% of the cases, monosomy or heterozygous deletion was detected, in 8% polysomy and in 1 SCC patient (2% of cases) an amplification was observed (**Table 2** and **Figure 1A**). None of the cases met criteria for copy number gain.

Next, we assessed PD-L1 mRNA expression by RNAish (**Table 2** and **Figure 1B**). Within tumor cells, expression was detected in 30 of 54 evaluable cases (56%) and in the stromal compartment in 30 of 52 evaluable cases (52%). PD-L1 mRNA expression within cells in the stroma was detected more frequently in SCC as compared to AC, 48 vs. 8% relatively (p < 0.05).

When protein expression was examined by IHC, a mean of 41% of tumor cells were PD-L1 positive and 53% were PD-L2 positive (**Table 2** and **Figures 1C, D**). Furthermore, stromal field cells were PD-L1 positive in 83% of the cases, whereas 39% of cells in the stromal fields were PD-L2 positive (**Figures 1C, D**). As compared to AC, cells in SCC stroma were more often PD-L1 positive (p = 0.044), as were tumor (p = 0.000) and stroma compartment cells (p = 0.005) for PD-L2, based on H-scores of 0 (i.e., negative) versus values exceeding 0 (i.e., positive) (**Table 2**).

### Expression and Distribution of PD-L1 and PD-L2 in Tumor Digests Based on Flow Cytometry Analyses

**Figure 2:**
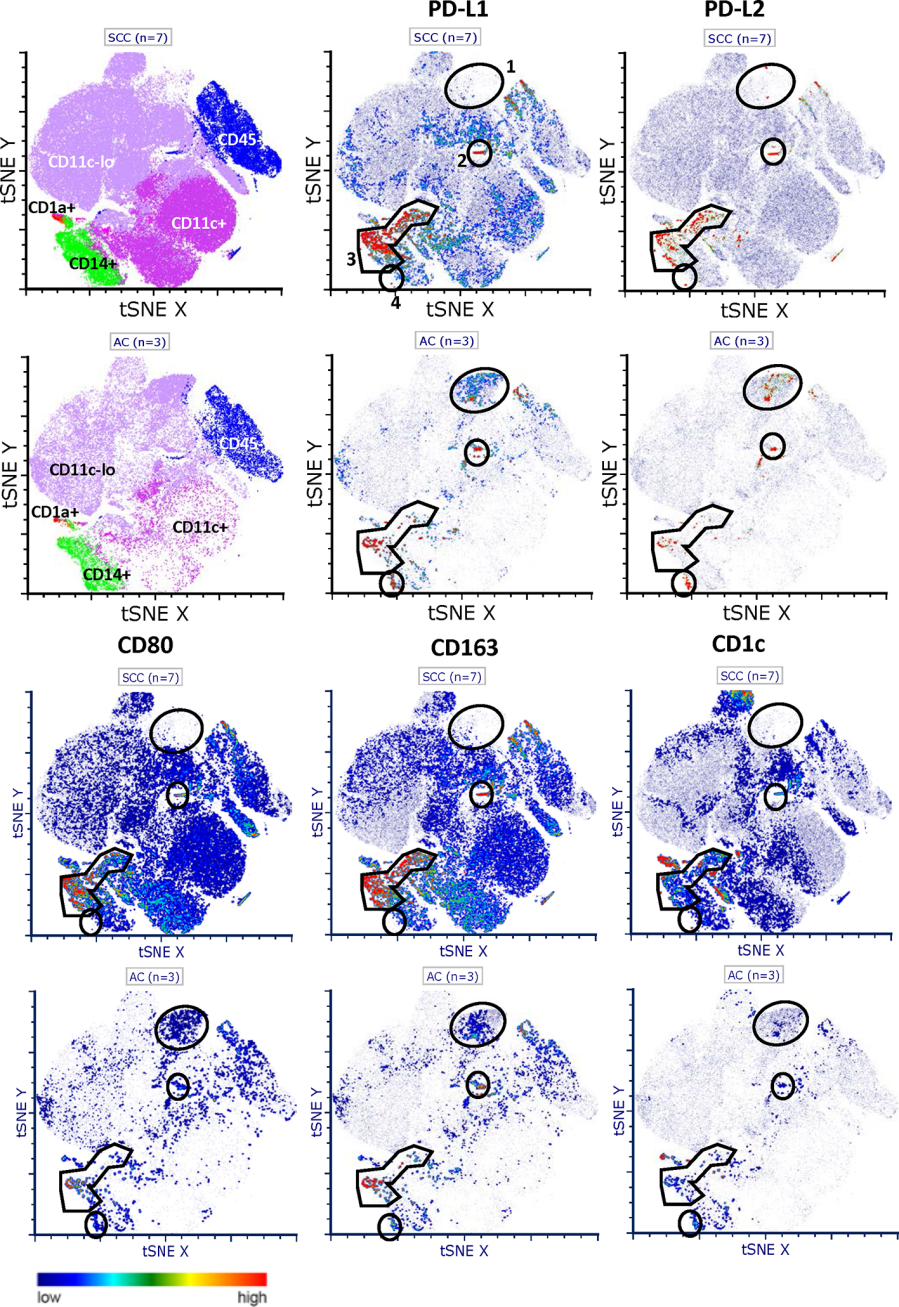
t-SNE plots of PD-L1 and PD-L2 expression and distribution in SCC and AC. Comprehensive flowcytometric analysis on tumor digests of SCC (n = 7, top rows) and AC (n = 3, bottom rows). t-SNE analysis shows four PD-L1 and PD-L2 co-expressing macrophage-like populations, based on CD14 or CD163 positivity and low or absent CD1c expression. No. 1) a large population over-represented in AC with relatively high levels of PD-L1 and PD-L2 and low levels of CD80. No. 2) a small PD-L1^+^PD-L2^+^CD80^−^CD163^+^ M2-like macrophage population present in both SCC and AC. No. 3) most frequent population with high levels of both PD-L1 and PD-L2. In SCC only, high co-expression of CD80. No. 4) a small PD-L1^+^/PD-L2^+^ population in AC with low levels of CD80.

In order to gain more insight in the expression of PD-L1 and PD-L2 in relation to CD80 on infiltrating immune cell populations, we performed additional comprehensive flow cytometric analyses on tumor digests of 7 SCC and 3 AC. t-SNE analysis revealed four PD-L1 and PD-L2 co-expressing macrophage-like populations (i.e., CD14 or CD163 positive and low or absent expression of CD1c), designated 1 through 4 (see **Figure 2**). A very small PD-L1^+^PD- L2^+^CD80^-^CD163^+^ M2-like macrophage population (no. 2) was present in both SCC and AC. The most frequent population (no. 3) expressed high levels of both PD-L1 and PD-L2. Remarkably, in SCC this population showed equally high co-expression levels of CD80, whereas in AC CD80 levels were lower, consistent with a more immune suppressive M2-like phenotype. Similarly, a larger (no. 1) and smaller (no. 4) population were over-represented in AC and expressed relatively high levels of PD-L1 and PD-L2 and low levels of CD80. In conclusion, PD-L1 and PD-L2 co-expressing macrophage-like populations appear to be more frequent in SCC, but in that sub-type, these macrophages are marked by co-expression of CD80. In contrast, in AC, relatively more infiltrating macrophages express PD-L1 and PD-L2 with absent or low-level expression of CD80.

### Comparison of DNA, mRNA, and Protein Expression of PD-L1 and PD-L2

**Figure 3:**
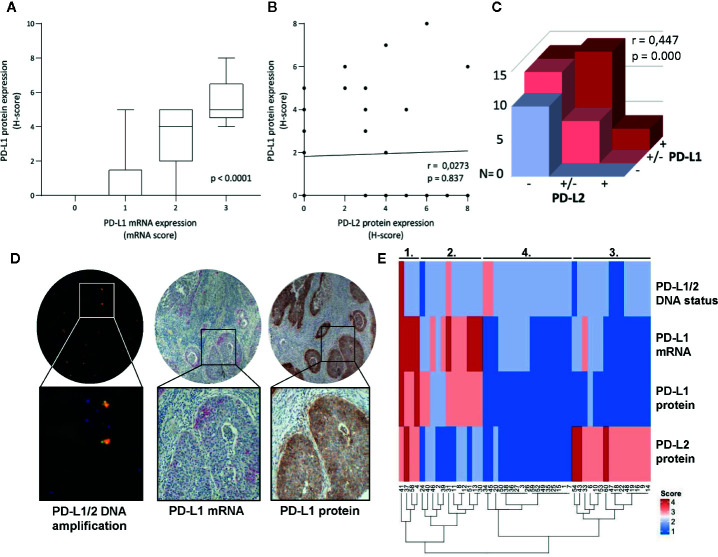
Comparison of DNA status, mRNA- and protein expression of PD-L1/PD-L2. **(A)** Higher mRNA expression levels of PD-L1 in tumor cells associated with higher PD-L1 protein expression on tumor cells as assessed by the H-score. **(B)** No correlation was found between PD-L1 and PD-L2 protein expression on tumor cells as assessed by the H-score (Spearman r = 0.0273, p = 0.837). **(C)** Correlation between PD-L1 and PD-L2 protein expression on cells in stroma (Spearman r = 0.447, p = 0.000). **(D)** Microscopic images of the case with PD-L1/PD-L2 DNA amplification and accordingly high mRNA PD-L1 expression (in red, >10 dots/tumor cell) and high PD-L1 protein expression (in brown, H-score = 8). **(E)** Heatmap cluster analysis based on PD-L1/PD-L2 DNA status, PD-L1 mRNA-, PD-L1 protein-, and PD-L2 protein expression on tumor cells (n = 48).

Cases with high PD-L1 protein expression had also higher mRNA expression (p < 0.0001, **Figure 3A**). No correlation for PD-L1 protein and PD-L2 protein expression on tumor cells was observed (**Figure 3B**, p = 0.837), but on stromal field cells, a correlation was observed (**Figure 3C**, Spearman r = 0.44, p < 0.001). The only case with amplification of both CD274 and PDCD1LG2 loci had correspondingly high mRNA expression of PD-L1, as well as strong PD-L1 and high PD-L2 protein expression within the tumor fields (**Figure 3D**). To visualize the co-expression of PD-L1/PD-L2 of all patients for whom all parameters were available (n = 48), a heat map was created based on copy number and mRNA/protein expression in the tumor and groups could be divided into four clusters: 1) patients with high expression of both PD-L1 and PD-L2; 2) patients with intermediate PD-L1-expressing samples and without or low PD-L2 expression; 3) patients without or with low PD-L1 expression but with high PD-L2 expression; and 4) patients without or with low expression of both PD-L1 and PD-L2 (**Figure 3E**). Interestingly, all four patients in cluster 1 (high expression of both PD-L1 and PD-L2) had cervical cancer of the SCC subtype and most of the patients with AC were grouped together in cluster 4 (low expression of both PD-L1 and PD-L2) (**Table 3**). We deemed the cohort too small to perform meaningful subgroup analysis based on clinicopathological parameters.

**Table 3:** Histological subtype differences between heatmap clusters.

Clusters heatmap		Total	Histological type			p-value^a^
		N (%)	SCC	AC	ASCC	
**1**	PD-L1^hi^/PD-L2^hi^	4 (8)	4 (11)	0	0	0.007
**2**	PD-L1^hi^/PD-L2^lo^	12 (25)	9 (25)	3 (27)	0
**3**	PD-L1^lo^/PD-L2^hi^	15 (31)	15 (42)	0	0
**4**	PD-L1^lo^/PD-L2^lo^	17 (36)	8 (22)	8 (73)	1 (100)
**Total**		48	36	11	1

SCC, squamous cell carcinoma; AC, adenocarcinoma; ASCC, adenosquamous cell carcinoma; hi, high; lo, low.

^a^χ^2^calculated only between SCC and AC, due to only 1 ASCC case. Underlined means statistically significant.

### Heterogeneity of PD-L1/PD-L2 Protein and PD-L1 mRNA Expression Within TMA Cores

**Figure 4:**
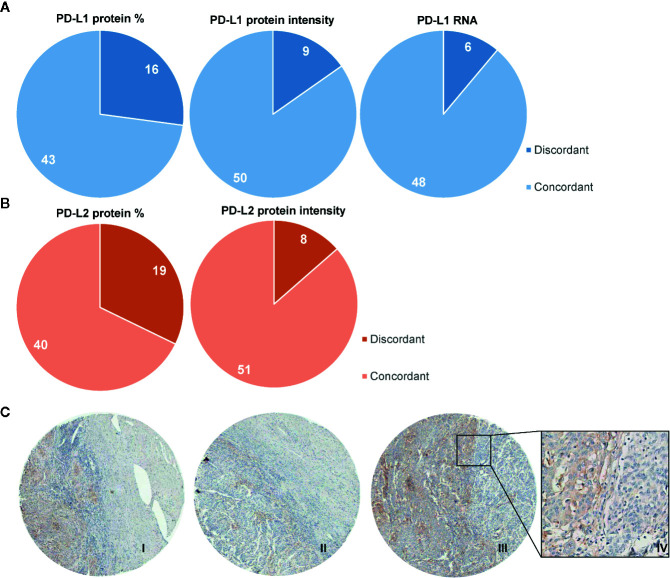
Heterogeneity of protein expression and mRNA within TMA cores. Diagrams showing the number of cases where discordant scores were given to TMA core biopsies derived from one patient. **(A)** In 16/59 patients, a different percentage of PD-L1 expression was observed, based on 5 categories (0: 0%–1%; 1: 1%–10%; 2: 11%–25%; 3: 26%–50%; 4: 51%–75%; 5: 76%–100%; left panel). 9/59 patients were scored with different intensities of PD-L1, based on 4 categories (0: negative; 1: weak; 2: clear; 3: strong; middle panel). In 6/54 patients different scores were given for mRNA PD-L1, based on 5 categories (0: no dots; 1: 1–3 dots per tumor cell in <10% of tumor fields; 2: 1–3 dots per tumor cell in ≥ 10% of tumor fields; 3: 4–10 dots per tumor cell; 4: ≥10 dots per tumor cell). **(B)** 19/59 patients had discordant scores for PD-L2 protein percentages on tumor cells. **(C)** 8/59 patients had discordant scores for PD-L2 protein expression based on intensity. Immunohistochemical example of discordant PD-L1 expression pattern (in brown) on tumor cells (I, II, and III with H-scores of 5, 3, and 6, respectively) with also a representative example of heterogeneity within one core (IV).

Next, we investigated the heterogeneity of the PD-L1/PD-L2 protein and mRNA PD-L1 expression on tumor cells by examining the scoring discrepancy between the cores per patient. Discordant scores for PD-L1 protein expression on tumor cells between cores from one patient were observed in 27% of cases, based on percentage of positive tumor cells, and in 15% of cases based on intensity of PD-L1 staining. Interestingly, with RNAish, PD-L1 heterogeneity was observed in only 11% of the cases (**Figure 4A**). Similarly to the PD-L1 IHC scores, in 32% of the samples discordant percentages of PD-L2 protein expression between cores were found (**Figure 4B**). Based on PD-L2 intensity, 14% of scores had different values between cores. An example of the heterogeneity of PD-L1 protein expression between cores within one patient is illustrated in **Figure 4C**-I-III. **Figure 4C**-IV also shows heterogeneity of PD-L1 expression within one core.

### TCGA-Derived Copy-Number and mRNA Sequencing Data

**Figure 5:**
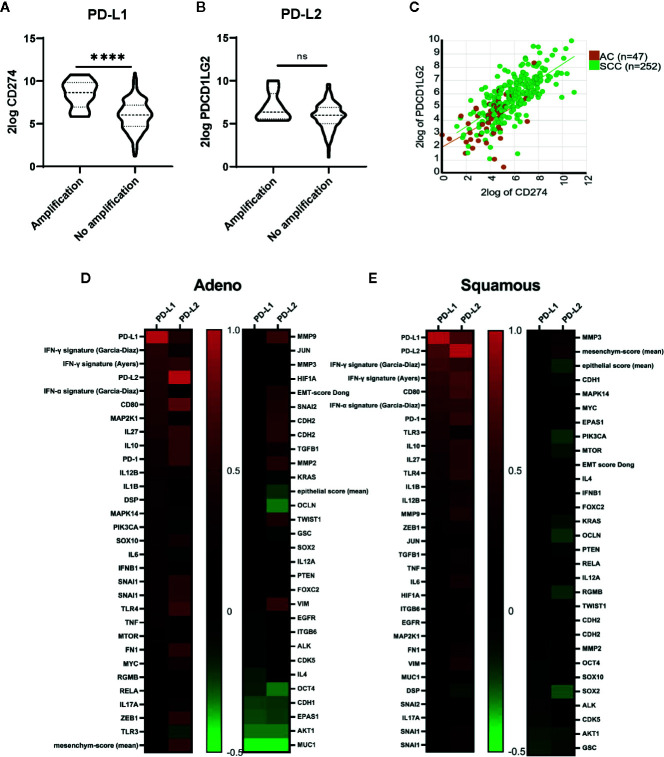
TCGA derived copy-number and mRNA sequencing data. **(A)** Higher levels of PD-L1 mRNA transcripts were found in patients with PD-L1 locus amplification (n = 8) compared with patients without amplification (n = 285). **(B)** No significant differences in PD-L2 mRNA expression were found for patients with amplification compared to those without amplification. **(C)** A strong correlation for PD-L1 and PD-L2 mRNA transcript levels was found (AC, r = 0.516, p < 0.001; SCC, r = 0.699, p < 0.0001). **(D)** Correlations (Pearson r-values) for genes described as regulators of PD-L1 and PD-L2 are shown for cervical adenocarcinoma. Gene signatures for IFNG signaling pathway (Garcia-Diaz) consisting of *IFNGR1*, *IFNGR2*, *STAT1*, *JAK1*, *JAK2*, *IRF9*, *STAT2*, and *STAT3*. IFNG response signature (Ayers): *IDO1*, *CXCL10*, *CXCL9*, *HLA-DRA*, *STAT1*, and *IFNG.* IFN-α signaling (Garcia-Diaz) signature: *IFNAR1*, *IFNAR2*, *JAK1*, *TYK2*, *STAT1*, *STAT2*, *STAT3*, and *IRF9* **(E)** Correlations (Pearson r-values) for genes described as regulators of PD-L1 and PD-L2 are shown for cervical squamous cell carcinoma. ****P ≤ 0.0001. ns, not significant.

To study transcriptional regulation of PD-L1/PD-L2 expression we used mRNAseq data from the TCGA database. Within the cervical cancer cohort, 8 of 293 evaluable patients (3%) had reported amplification of the PD-L1/PD-L2 locus. These cases, which were all SCC, had significantly higher levels of PD-L1 *(CD274*, p < 0.0001*)*, but not PD-L2 *(PDCD1LG2*, p > 0.05*)* mRNA expression compared to the SCC cases without amplification (**Figures 5A, B**). PD-L1 and PD-L2 mRNA levels showed a strong correlation in both histological subtypes (**Figures 5C–E**; AC, r = 0.516, p < 0.0001; SCC, r = 0.699, p < 0.0001). Both PD-L1 and PD-L2 showed a correlation with a canonical IFNG signaling pathway signature as described by Garcia-Diaz et al. (37, 38) consisting of *IFNGR1*, *IFNGR2*, *STAT1*, *JAK1*, *JAK2*, *IRF9*, *STAT2* and *STAT3* (p < 0.001 for PD-L1 for both AC, (r = 0.500), and SCC (r = 0.640); p < 0.0001 for PD-L2 in SCC (r = 0.567) and P = 0.03 for AC (r = 0.317) (**Figures 5D, E**). Also the IFN-γ response signature, which was associated with clinical response to PD-1 blockade as described by Ayers and colleagues (39), correlated with both PD-L1 and PD-L2 mRNA in AC (r = 0.55; p < 0.001; r = 0.57; p < 0.001 respectively) and in SCC (r = 0.58; p < 0.0001; r = 0.67; p < 0.001, respectively). This 6-gene signature consists of *IDO1*, *CXCL10*, *CXCL9*, *HLA-DRA*, *STAT1*, and *IFNG.* The canonical pathway for interferon alpha (IFN-α) signaling (consisting *IFNAR1*, *IFNAR2*, *JAK1*, *TYK2*, *STAT1*, *STAT2*, *STAT3*, and *IRF9*) showed a correlation with PD-L1 in AC (p <0.0001, r = 0.50) and a correlation for both PD-L1 (r = 0.51) and PD-L2 (r = 0.53) in SCC (p < 0.0001 for both) (37). Next to the interferon signatures, CD80 showed a strong correlation with PD-L1 and PD-L2 for both AC (r = 0.492 for PD-L1, r = 0.762 for PD-L2) and SCC (r = 0.537 for PD-L1, r = 0.645 for PD-L2) (**Figures 5D, E**; p < 0.0001 for all). Other genes that have been described (17) as regulators of PD-L1 showed less strong correlations with PD-L1 (see **Figures 5D, E**).

**Figure 6:**
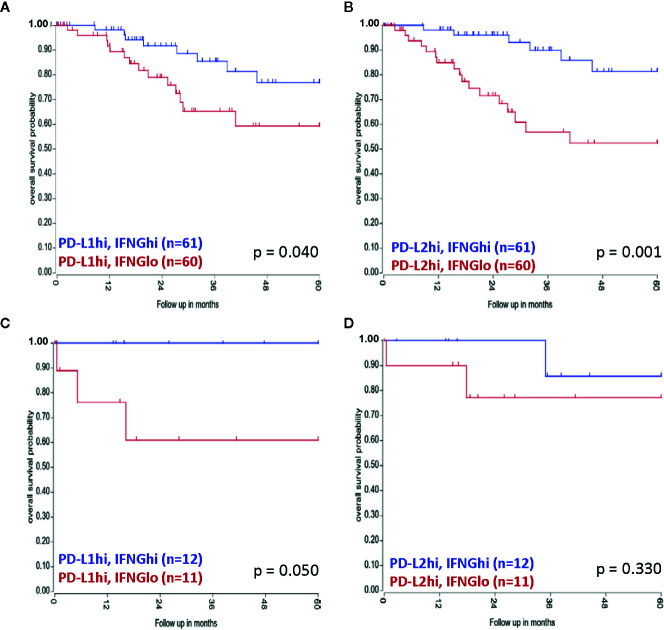
Combined TCGA mRNA transcript levels of PD-L1/PD-L2 and IFNG and associations with survival. **(A)** A survival advantage for SCC patients with PD-L1hiIFNGhi mRNA transcript levels was observed, as well as for SCC patients with **(B)** PD-L2hiIFNGhi transcript levels. For AC patients **(C)** PD-L1hiIFNGhi was linked to better prognosis, whereas **(D)** PD-L2hiIFNGhi was not associated with survival.

No associations for mRNA expression levels and/or amplification cases with patient survival were found for PD-L1 and PD-L2 (data not shown). However, combining transcript levels of IFNG with PD-L1 and PD-L2, a survival advantage for SCC patients with high PD-L1 or PD-L2 levels and high IFNG mRNA expression levels (PD-L1hiIFNGhi/PD-L2hiIFNGhi) was observed, compared to patients with PD-L1hiIFNGlo/PD-L2hi/IFNGlo, with median expression as cut-off (p = 0.040 for PD-L1 and p = 0.001 for PD-L2) (**Figures 6A, B**). Also in AC patients, the PD-L1hiIFNGhi group had a better overall survival than patients with PD-L1hiIFNGlo (p = 0.050) (**Figure 6C**). For PD-L2hi no significant difference in survival was observed in the AC patient cohort based on IFNG expression levels (p = 0.330) (**Figure 6D**). However, the AC patient group was small.

Next, we assessed which genes had higher expression in the IFNGlo compared with the IFNGhi group, to unveil other possible mechanisms responsible for PD-L1/PD-L2 expression in a microenvironment with low IFNG expression (**Supplementary Tables 1** and **2**). For SCC, KEGG (Kyoto Encyclopedia of Genes and Genomes) gene set analysis showed that in the PD-L1hiIFNGlo 90 genes were expressed at a significantly higher level than the PD-L1hiIFNGhi group (with p < 0.01 as cut-off). Thirty-four of these genes were in a KEGG gene set collection and were involved in various signaling pathways, most notably the Wnt and hedgehog signaling pathways and pathways regulating pluripotency of stem cells (**Supplementary Table 1**). In total, 29 genes had higher expression (with p > 0.01) in the PD-L2hiIFNGlo group compared to the PD-L2hiIFNGhi group. Only 11 of these genes were present in a KEGG gene set (**Supplementary Table 2**). In AC, no genes were found that were significantly deferentially expressed between the PD-L1hiIFNGhi and PD-L1hiIFNGlo group and the PD-L2hiIFNGhi and PD-L2hiIFNGlo group.

## Discussion

In this study, we report that mRNA expression of PD-L1 and protein expression of PD-L1 and PD-L2 are common in cervical cancer, while amplification of the PD-L1/PD-L2 locus is rare. Importantly, considerable PD-L1 heterogeneity was observed based on IHC protein expression, and we propose future investigations in the application of *in situ* PD-L1 mRNA expression as a novel biomarker. Publicly available TCGA mRNAseq and copy number data was used as a validation cohort and for additional exploratory analyses to uncover regulation mechanisms of PD-L1/PD-L2 expression in cervical cancer.

Patients with amplification of the PD-L1/PD-L2 locus have high response rates to immune checkpoint blockade, but amplification incidence in solid tumors is generally very low (< 1%) (40). Amplification was indeed rare in our cohort (2%), in contrast to the report of Howitt *et al.* where the prevalence was 23% (31). This discrepancy might be explained by the CNA scoring method used in the latter study, where cases were classified according to the highest observed genetic abnormality by FISH analysis; even cases in which only 2% of evaluated cells showed amplification were classified as “amplification”. Detection of a higher amount of probe signals in a minority of cells can however be a result of overlapping cells on Z-stack images and/or background signals. In our study, cases were classified by the most abundantly present genetic abnormality, in concordance with the conventional diagnostic approach (29). Our results are comparable with other reports, showing amplification rates of 2.7% (40) and were confirmed by additional TCGA analysis (3% amplification rate). The one amplification case in our cohort showed a high level of PD-L1 mRNA and protein expression. Correspondingly, PD-L1 mRNA levels were significantly higher in amplification cases in the TCGA cohort. Also in lymphomas, increased PD-L1 expression was detected in cases with 9p24.1 amplification (30, 41, 42).

Previous studies have shown a strong correlation between PD-L1 mRNA expression and protein expression in lung cancer, lymphoma and neuroendocrine tumors (43–47). PD-L1 mRNA expression and PD-L1 protein expression were also strongly correlated in the present cohort (p < 0.001). However, still in 20% of the cases, only PD-L1 mRNA expression and no PD-L1 protein expression was detected. This heterogeneity in protein expression could be caused by post-translational modifications (48). RNAish detection of PD-L1 mRNA might therefore be more sensitive when compared to PD-L1 protein expression by IHC and might serve as a better predictive biomarker for immune checkpoint therapy, specifically as in various cancer types PD-L1 negative patients did show response to PD-L1/PD-1 checkpoint inhibition (49).

There is a need for consensus on sampling, staining and scoring procedures concerning detection of PD-L1/PD-L2 protein expression by IHC to improve reproducibility of studies and confirm inter-study results. Here, we used the SP142 antibody clone for PD-L1 protein detection. Huang *et al.* compared two clones (SP142 and SP263) commonly used for PD-L1 IHC, and showed SP263 staining to be easier to evaluate, whereas SP142 was more strongly correlated with survival rates in diffuse large B-cell lymphoma (50). However, compared with other clones, such as 22C3, 28-8, and SP263, the SP142 antibody has been described to be least sensitive for tumor cell expression (51). In a previously published study on cervical cancer though, we have had good experience with this clone for the detection of PD-L1 on both tumor cells as well as on immune cells (24), and as well, the SP142 clone has been approved by the FDA as companion diagnostic for atezolizumab (anti-PD-L1) treatment in several other cancer indications (52, 53). Furthermore, we used an H-score to evaluate PD-L1 and PD-L2 expression. Also a tumor proportion score (TPS) and combined positive score (CPS) have been developed and are used frequently. The TPS is defined as the percentage of tumor cells that express PD-L1 and uses thresholds of 1%, 10%, and 50% (54). This score does not take the expression of PD-L1 on cells in stromal fields into account. The CPS is a ratio of all cells that express PD-L1 (tumor cells and immune cells) to the number of all tumor cells (55). In cervical cancer, pembrolizumab was approved by the FDA for treatment of patients with a CPS ≥ 1, with the PD-L1 IHC 22C3 clone (26). However, consensus on universal staining and scoring methods for PD-L1/PD-L2 expression has not been reached. In this study, we evaluated different cores from one tumor to determine heterogeneity between biopsies. The high number of samples with discordant scores for PD-L1 (27%) and PD-L2 (32%) in our study demonstrates their expression heterogeneity in cervical cancer, also shown in other tumor types (56–59). Core biopsies for patient selection can be punched in a negative area of a (partly) positive tumor, which consequently leads to false-negative results. This might explain controversial response rates regarding PD-L1 patient selection based on biopsies. The use of a TMA for this study also has its limitations when compared to whole section analysis (60). For instance, PD-L1 expression patterns (diffuse vs. marginal), which have been shown to be pivotal for patient survival, cannot be detected in these small core biopsies (24, 61) since the tumor-stroma interface is not always included during construction of the TMA. In the future, PD-L1 PET-CT imaging may present as a good alternative to non-invasively determine the PD-L1 status of tumors and/or metastases in order to overcome false-negative results due to tumor heterogeneity (62).

Here, we found no correlation between PD-L1 and PD-L2 ligand expression by tumor cells, in concordance with findings in lung and esophageal AC (63, 64). The co-expression of PD-L1 and PD-L2 on myeloid infiltrating cells (see **Figure 2**) suggests that expression of both proteins may be induced in the same manner, for instance by IFNG signaling, while expression on tumor cells might be caused by different mechanisms (65, 66). It was shown that both PD-L1 expression on tumors cells alone or on immune cells alone, were related to responses in patients treated with anti-PD-L1 therapy in NSCLC (65). In melanoma patients treated with ipilimumab and nivolumab, the expression of PD-L1 on immune-infiltrating cells had an even stronger predictive value, than the expression of PD-L1 on tumor cells (13), warranting further research into the role of PD-L1 and PD-L2 on stromal compartment cells versus tumor cells. Of note, a recent report showed that interactions *in cis* of co-expressed PD-L1 and CD80 led to the inactivation of PD-L1 but the maintained co-stimulatory ability of CD80 (67). In this regard, it is of particular interest that PD-L1 on macrophages in SCC was mostly co-expressed with CD80, in contrast to AC. Thus, although PD-L1-expressing macrophage-like cells are more frequent in SCC, in AC they might be more effective in terms of T cell suppression, due to the absence of CD80 on their cell surface, and as such possibly also more predictive for PD-(L)1 blockade efficacy.

In this study, analyses in subcohorts based on histological subtypes (SCC vs. AC) show higher PD-L1 mRNA, PD-L1 and PD-L2 protein expression in SCC when compared to AC. The origin and regulation of PD-L1/PD-L2 expression might be accordingly different in these histological subtypes. Previous studies also showed that expression of PD-L1 is more prevalent in cervical SCC as compared to cervical AC (24, 68) and also in the current study, by visualizing the distribution of PD-L1 and PD-L2 using high dimensional t-SNE analyses, it was clearly shown that AC tumors and their infiltrates express both PD-L1 and PD-L2 to a lesser extent, although, as discussed above, with generally lower levels of co-expressed CD80. Therefore, it is important for future trials investigating immune checkpoint therapy to incorporate histological subcohort analysis.

PD-L1 protein expression is currently used as a predictive biomarker for checkpoint therapy in cervical cancer with suboptimal results. As our cohort and the TCGA cohort did not contain cervical cancer patients that were treated with checkpoint therapy, we were not able to evaluate the PD-L1/PD-L2 predictive potential. Because our cohort was limited in size, consisted of mainly early stage cervical cancer (72%) and consequently had low 5 year recurrence- (n = 8/60) and death rates (n = 4/60), we performed survival analyses based on the TCGA database. We found that patients with high PD-L1 or PD-L2 mRNA expression and high IFNG signaling activity, consistent with an ongoing T-cell response inducing PD-L1 expression, had better overall survival compared to patients with high PD-L1 and low IFNG expression. In the latter group, PD-L1 upregulation might be explained by other (not immune-related) factors; for instance associated with oncogenesis and stem cell-related aberrant Wnt and Hedgehog signaling pathways (69), as we observed in the SCC TCGA cohort. The plethora of factors, at the genetic, epigenetic, transcriptional, translational and post/translational level that have been described to influence PD-L1 expression is impressive and underlines the complexity of the regulation of PD-L1 expression (16, 17).

Our observations show that amplification of the PD-L1/PD-L2 gene in cervical cancer patients is a rare event, which makes it an unsuitable biomarker of response to checkpoint inhibition. RNAish appears to be the most sensitive and consistent detection method. Future research, including a larger patient cohort, should evaluate whether RNAish could serve as a better biomarker than IHC detection. Importantly, we conclude that interferon signaling is the major cause of PD-L1 expression in cervical cancer and is correlated with improved survival. Evaluation of interferon signaling in conjunction with PD-L1/PD-L2 expression for prediction of clinical response to immune checkpoint therapy should be considered.

## Data Availability Statement

The raw data supporting the conclusions of this article will be made available by the authors on request.

## Ethics Statement

The studies involving human participants were reviewed and approved by Institutional Review Board of the Netherlands Cancer Institute. Written informed consent for participation was not required for this study in accordance with the national legislation and the institutional requirements.

## Author Contributions

Conception and design: JR, LO, and EJ. Development of methodology: MR, MB, and EJ. Acquisition of data (provided animals, acquired and managed patients, provided facilities, etc.): JR, LO, AH, SS, MR, NT, HZ, GK, MB, TG, and EJ. Analysis and interpretation of data (e.g., statistical analysis, biostatistics, computational analysis): JR, LO, AH, EJ, and TG. Writing, review, and/or revision of the manuscript: JR, LO, AH, EJ, and TG. Administrative, technical, or material support (i.e., reporting or organizing data, constructing databases): JR, AH, SS, and MB. Study supervision: GK, TG, and EJ. All authors contributed to the article and approved the submitted version.

## Funding

This work was supported by research grants from the Stichting VUmc-CCA (CCA2015-3-06) and the Louise Vehmeijer Foundation.

## Conflict of Interest

The authors declare that the research was conducted in the absence of any commercial or financial relationships that could be construed as a potential conflict of interest.

## Supplementary Material

Supplementary Table 1 SCC - KEGG gene set analysis.

Supplementary Table 2 AC - KEGG gene set analysis.
