# Supplementary material for: PD-L1 and PD-L2 Expression in Cervical Cancer: Regulation and Biomarker Potential
Source: Front Immunol. 2020 Dec 17;11:596825. doi: 10.3389/fimmu.2020.596825 (PMC7793653; doi:10.3389/fimmu.2020.596825)
Supplement: Supplementary Table 1 — SCC - KEGG gene set analysis. [file DataSheet_1.pdf]

HUGO	R	pval
ADAMTS12	0.325	6.39e-03
FRK *kinase,sign transd*	0.432	3.22e-05
MFN1 *membrane*	0.313	9.85e-03
IPO9	0.430	3.49e-05
ADAM23 *development,membrane*	0.313	9.82e-03
CAMSAP2	0.425	4.79e-05
HECTD1	0.313	9.77e-03
PUM1	0.410	1.11e-04
PLEKHG6 *sign transd*	0.410	1.14e-04
SUN1	0.407	1.36e-04
SH3PXD2B	0.400	2.01e-04
MAP3K4 *kinase,sign transd*	0.399	2.08e-04
PLEKHA1 *membrane*	0.387	3.96e-04
LRP6 *development,membrane ,sign transd*	0.314	9.59e-03
ZNF704	0.384	4.63e-04
SHISA2	0.383	4.83e-04
FAM172A	0.314	9.40e-03
PRICKLE1 *membrane*	0.383	4.85e-04
UBTD2	0.378	6.20e-04
SHANK1	0.378	6.39e-04
PRND *membrane*	0.376	6.84e-04
LRP4 *membrane*	0.371	8.92e-04
PCLO *membrane*	0.367	1.09e-03
CNKSR3 *membrane,sign transd*	0.315	9.20e-03
LRRC17	0.315	9.20e-03
TULP3 *membrane,sign transd*	0.315	9.20e-03

B3GNT5 *development,membrane*	0.315	9.17e-03
VGLL4	0.365	1.16e-03
TOM1L1 *membrane*	0.365	1.18e-03
TANC1	0.316	9.05e-03
ZC3HAV1L	0.316	9.05e-03
PPFIBP1 *membrane*	0.361	1.40e-03
GALNT13 *membrane*	0.360	1.44e-03
SERTAD4	0.360	1.47e-03
ACPP	0.360	1.48e-03
PSD3 *sign transd*	0.359	1.55e-03
MMP16 *drugtarget,membrane*	0.358	1.58e-03
FZD7 *development,membrane ,sign transd*	0.358	1.59e-03
KLHL42	0.358	1.61e-03
ACVR1 *apoptosis,cell cycle ,development, diff ,drugtarget,kinase ,membrane,sign transd*	0.358	1.63e-03
TACC2	0.356	1.73e-03
TFRC *membrane*	0.353	2.02e-03
ZRANB1	0.353	2.05e-03
PCYT1B *drugtarget*	0.350	2.26e-03
CNTN1 *membrane,sign transd*	0.349	2.39e-03
RASAL2 *sign transd*	0.319	8.08e-03
SHROOM4	0.349	2.40e-03
FZD6 *development,membrane*	0.347	2.59e-03

,sign transd		
DSTYK	0.319	7.96e-03
NHSL1	0.347	2.61e-03
SBF2 *development,membrane*	0.319	7.84e-03
RAET1L *membrane*	0.345	2.91e-03
IGFBP2	0.344	2.92e-03
DCUN1D1	0.342	3.20e-03
DIP2B	0.341	3.35e-03
PRICKLE2 *membrane*	0.341	3.38e-03
CTTNBP2	0.340	3.51e-03
USP54	0.339	3.58e-03
DIRAS1 *membrane,sign transd*	0.320	7.70e-03
PLD1 *drugtarget,membrane ,sign transd*	0.338	3.72e-03
ITGAV *membrane,sign transd*	0.338	3.75e-03
PCDH19 *membrane*	0.338	3.79e-03
SERTAD4-AS1	0.336	4.03e-03
ATP8B1 *membrane*	0.336	4.04e-03
CDS1 *membrane,sign transd*	0.336	4.12e-03
PTPRF *membrane,sign transd*	0.335	4.31e-03
KIAA1715 *development,membrane*	0.334	4.52e-03
ULBP3 *membrane*	0.332	4.76e-03
MTSS1L	0.321	7.28e-03
ITGB5 *development,membrane ,sign transd*	0.331	4.92e-03

PRKG2 *kinase,sign transd*	0.331	4.92e-03
PLEKHG1 *sign transd*	0.331	4.92e-03
TMEM47 *membrane*	0.331	4.94e-03
AKR1C2 *drugtarget*	0.331	4.99e-03
CTDSPL2	0.330	5.11e-03
SC5D	0.322	7.12e-03
MFSD11	0.330	5.23e-03
ADGRA3	0.329	5.38e-03
FREM2 *development,membrane*	0.328	5.64e-03
APCDD1L *membrane*	0.328	5.67e-03
XKRX *membrane*	0.328	5.68e-03
GNAL *sign transd*	0.323	6.78e-03
ATP2B4 *membrane*	0.327	5.91e-03
WNT5A *development,sign transd*	0.323	6.76e-03
PLEKHA5	0.327	5.99e-03
SMO *apoptosis,development ,diff,membrane ,sign transd*	0.324	6.60e-03
KIAA1549L	0.326	6.04e-03
INPP5A *membrane*	0.326	6.22e-03
ZAK *apoptosis,cell cycle ,diff,kinase ,sign transd*	0.326	6.24e-03
LGR5 *membrane,sign transd*	0.324	6.48e-03

Set	R#	No of genes	P-value	Genes
over-representation Basal_cell_carcinoma	55	4	9.9e-10	, FZD6, FZD7,
over-representation Wnt_signaling_pathway	140	6	1.0e-08	, FZD6, FZD7,
over-representation Proteoglycans_in_cancer	200	6	6.6e-06	, FZD6, FZD7,
over-representation Signaling_pathways_regulating_pluripotency_of_stem_cells	141	4	9.1e-04	, ACVR1, FZD6, FZD7,
over-representation Glycerophospholipid_metabolism	94	3	3.3e-03	, CDS1, PCYT1B,
over-representation Melanogenesis	100	3	5.0e-03	, FZD6, FZD7,
over-representation Hedgehog_signaling_pathway	50	2	0.01	, SMO, WNT5A
over-representation Pathways_in_cancer	397	6	0.01	, FZD6, FZD7,
over-representation Cell_adhesion_molecules__CAMs_	139	3	0.03	, CNTN1, ITGAV,
over-representation Phagosome	150	3	0.04	, ITGAV, ITGB5,
