# Supplementary material for: PD-L1 and PD-L2 Expression in Cervical Cancer: Regulation and Biomarker Potential
Source: Front Immunol. 2020 Dec 17;11:596825. doi: 10.3389/fimmu.2020.596825 (PMC7793653; doi:10.3389/fimmu.2020.596825)
Supplement: Supplementary Table 2 — AC - KEGG gene set analysis. [file DataSheet_2.pdf]

HUGO	R	pval	Pres
RBM24	0.323	7.90e-03	116
APCDD1L r	0.385	5.05e-04	116
PPP6R3	0.375	7.99e-04	121
SHANK1	0.374	8.52e-04	92
CA12 drugta	0.367	1.20e-03	121
CTTN	0.362	1.51e-03	121
CAMSAP2	0.357	1.84e-03	121
KCND2 mem	0.356	1.93e-03	121
DHCR7 dev *,drugtarget ,membrane*	0.355	2.08e-03	121
SHISA2	0.352	2.38e-03	120
ARAP3	0.319	9.17e-03	121
ENDOD1	0.350	2.54e-03	121
FOXL1 TF,c *,transcripti on regulator Act*	0.319	9.06e-03	121
SRPX2	0.346	3.00e-03	121
SUN1	0.320	8.92e-03	121
TOM1L1 m	0.320	8.91e-03	121
LRP4 memb	0.346	3.02e-03	121
PTHLH dev *,sign transd*	0.344	3.37e-03	121
FRK kinase,	0.341	3.74e-03	121
GALNT5 m	0.336	4.52e-03	115

SYT7 memb	0.335	4.76e-03	121
ADAMTS12	0.332	5.36e-03	120
MMP16 dru	0.331	5.68e-03	116
ZAK apopto *,diff,kinase ,sign transd*	0.329	6.05e-03	121
PCDH10 me	0.322	8.19e-03	96
NPBWR1 m	0.328	6.30e-03	93
TACC2	0.322	8.07e-03	121
MAP3K4 ki	0.326	7.07e-03	121
MSRB3	0.323	7.98e-03	121

set	R#	#	_p_value_	Genelist
over-representation Nitrogen_metabolism		17	1 4.8e-03	, CA12
over-representation Tight_junction		137	2 6.8e-03	, CTTN, ZAK
over-representation Steroid_biosynthesis		20	1 0.01	, DHCR7
over-representation Mucin_type_O_Glycan_biosynthesis		30	1 0.04	, GALNT5
